# Hox-Positive Adult Mesenchymal Stromal Cells: Beyond Positional Identity

**DOI:** 10.3389/fcell.2020.00624

**Published:** 2020-07-31

**Authors:** Maria Kulebyakina, Pavel Makarevich

**Affiliations:** ^1^Department of Biochemistry and Molecular Medicine, Faculty of Medicine, Lomonosov Moscow State University, Moscow, Russia; ^2^Laboratory of Gene and Cell Therapy, Institute for Regenerative Medicine, Lomonosov Moscow State University, Moscow, Russia

**Keywords:** *Hox* genes, mesenchymal stem/stromal cells, postnatal morphogenesis, regeneration, stroma, tissue renewal

## Abstract

Homeotic genes *(Hox)* are universal regulators of the body patterning process in embryogenesis of metazoans. The *Hox* gene expression pattern (*Hox* code) retains in adult tissues and serves as a cellular positional identity marker. Despite previously existing notions that the *Hox* code is inherent in all stroma mesenchymal cells as a whole, recent studies have shown that the *Hox* code may be an attribute of a distinct subpopulation of adult resident mesenchymal stromal cells (MSC). Recent evidence allows suggesting a “non-canonical” role for *Hox* gene expression which is associated with renewal and regeneration in postnatal organs after damage. In tissues with high regenerative capacity, it has been shown that a special cell population is critical for these processes, a distinctive feature of which is the persistent expression of tissue-specific *Hox* genes. We believe that in the postnatal period Hox-positive subpopulation of resident MSC may serve as a unique regenerative reserve. These cells coordinate creation and maintenance of the correct structure of the stroma through a tissue-specific combination of mechanisms. In this article, we summarize data on the role of resident MSC with a tissue-specific pattern of *Hox* gene expression as regulators of correct tissue reconstruction after injury.

## Introduction

Homeotic genes (designated as *Hox* in mice and *HOX* in humans) is a family of homeotic genes encoding transcription factors known to function as master regulators of cell identity and fate during embryonic development of vertebrates. In mammals, *Hox* genes regulate patterning of the embryo along the body axis ranging from the anterior boundary of hindbrain till the tail end of the body. *Hox* genes are highly conserved among species and encode a set of proteins that share a high degree of structural similarity. In mammals, 39 *Hox* gene family members are organized into four clusters labeled A, B, C, and D – each cluster located on a separate chromosome – namely 7, 17, 12, and 2, respectively.

Each complex occupies a similar position in the harboring chromosome and its genes are numbered ranging from 1 to 13 according to their location from 3′ to 5′ terminus. However, not each group has all 13 genes, – some numbers may be missing (*e.g.*, *Hoxd* subgroup has nine genes). Genes of the same number (*e.g.*, *Hoxa9*, *Hoxb9*,*Hoxc9*, and *Hoxd9*) represent paralogues and demonstrate two unique features of *Hox* genes:

• *Spatial collinearity* which means that in the embryo their expression is activated in anteroposterior direction in the same order they are located on the chromosome, starting from 3′. For example, genes of paralogous group 3 are expressed posteriorly to genes of group 2 and all the way further to group 13.
• *Temporal collinearity* which means that during development 3′ genes are expressed earlier than 5′ genes. In vertebrates limb development is determined by posterior *Hox* genes (*Hox9-13*) reflecting the principle of collinearity – indeed, the development of distal body parts correlates with expression of “late-numbered” *Hox* genes. *E.g.*, within a developing limb *Hox9* genes are expressed in the most proximal portion while *Hox13* genes – in the most distal.

In adult organism cells can retain expression of *Hox* genes to form the distinct *Hox* expression pattern known as “*Hox* code” that strictly matches the *Hox* spectrum this part of the body demonstrated during embryonic development (Kmita and Duboule, 2003; Lappin et al., 2006; Svingen and Tonissen, 2006). *Hox* expression patterns are formed at the stage of mesoderm segmentation and subsequently remain unchanged reflecting positional affiliation of cells (Chang et al., 2002; Ackema and Charité, 2008; Picchi et al., 2013). *Hox* code is maintained throughout life in both – stromal and parenchymal cells, but the functional role of postnatal *Hox* gene expression in stroma remains highly enigmatic.

Further, we shall present an overview that will guide to a putative role of postnatal *Hox* expression in subpopulations of stromal cells as a feature required for tissue repair after damage in mammals (including human).

## Redundancy of *Hox* Genes and Structural Anomalies Caused by Their Disruption

From the functional point of view, Hox proteins are DNA-binding transcription factors that can act as an activator for certain target genes and a repressor for others. The specificity of Hox transcription factors binding to a DNA sequence is determined by protein cofactors from other families (Moens and Selleri, 2006). The main targets of Hox are proteins involved in chromatin remodeling and transcription factors which in summary suggests a major role of Hox in the regulation of genome expression through epigenetic and transcriptional control.

Thus, it is no surprise that a critical role of *Hox*-encoded proteins has been established in a great variety of cellular functions: cell growth, differentiation, migration, invasion, adhesion, etc. (Seifert et al., 2015). At the same time known regulators of *Hox* gene expression are scarce yet recent data suggests that *Hox* expression during embryogenesis is regulated by BMP, Wnt, and retinoic acid pathways. In the adult organism, several *Hox* regulators are found including vitamin D and steroid hormones which shall be discussed in detail below (Seifert et al., 2015; Du and Taylor, 2015).

Despite *Hox* genes’ pivotal role in embryo patterning, loss of function in a single *Hox* gene does not always lead to body structural malformation. This matches the existence of paralogous groups mentioned above although the molecular functions of genes among paralogous groups do not fully overlap. In other words, *Hox* genes are characterized by *functional redundancy*.

To date, several dozen mouse strains have been created carrying knockouts of different *Hox* genes. Many of these strains demonstrate profound inborn defects of organs and tissues while some (*e.g.*, with mutant *Hoxc5* or *Hoxa7*) do not display any developmental anomalies. A comprehensive analysis of deviations characteristic for these knockout mice strains can be found in the review by Quinonez and Innis (2014).

In humans, a dozen of developmental anomalies caused by mutations in *HOX* family genes have been described (Quinonez and Innis, 2014). For example, patients with a *HOXA2* gene mutation have microtia, a shortened and narrowed auditory tunnel and cleft palate (Alasti et al., 2008); hereditary hand-food-genital syndrome is a result of *HOXA13* mutations as well (Mortlock and Innis, 1997; Goodman et al., 2000).

## Postnatal Expression of *Hox* in Stromal Cells: Memorizing Location and Supporting Regenerative Potency

Stromal cells of mesodermal origin, such as mesenchymal stromal cells (MSC), fibroblasts, smooth muscle cells (Chi et al., 2007), and preadipocytes (Gesta et al., 2006), have a mechanism to memorize their topographic location in the form of their *Hox* code.

Indeed, fibroblasts isolated from topographically different body regions are characterized by a stable *Hox* code reflecting initial localization (Chang et al., 2002; Ackema and Charité, 2008). Moreover, the pattern of *Hox* gene expression in the postnatal period resembles that during development. For example, *Hox* codes in fibroblasts from different tissues coincide with *Hox* codes of mesoangioblasts of respective somites during embryogenesis (Ackema and Charité, 2008).

Lineage tracing data shows that in the postnatal period certain Hox-positive MSC (for example, expressing *Hoxa11*) do not arise from Hox-negative ones, but originate from pre-existed mesoangioblasts (Pineault et al., 2019). Thus, the expression of *Hox* genes in tissue-resident MSC does not seem to “turn on” in the postnatal period but rather remains unrepressed in some cells after birth. This is consistent with a confirmed postulate that in embryonic development *Hox* genes are repressed irreversibly and recapitulation of their expression seems impossible under physiological conditions (Wang et al., 2009). In this case, MSC inhere *Hox* code of their progenitors and retain it throughout life. This data supports a predisposition that in the postnatal period, tissues may contain subpopulations of Hox-positive MSC originating from embryonic progenitors and retaining their *Hox* code.

The *Hox* code of MSC reflects not only their location and harbor within the body but also serves as a “fingerprint” of tissue from which the cells were isolated. Hox-positive status is a very stable characteristic of a cell: it persists *in vitro* after prolonged culture, during differentiation, and in presence of soluble factors secreted by cells with another *Hox* code (Ackema and Charité, 2008). Expression of *Hox* in MSC is strongly resistant to the influence of exogenous factors (soluble molecules, hypoxia, stress, cell-to-cell contacts, etc.).

It was shown (Leucht et al., 2008) that the *Hox* code in stem cells is preserved even after transplantation to a Hox-negative organ or tissue within the host. In contrast, Hox-negative cells can adopt the *Hox*-code from the Hox-positive environment and this may occur after their heterotopic transplantation, as well as *in vitro* after co-culture with Hox-positive cells. Human unrestricted somatic stem cells (USSC) isolated from cord blood are characterized by the absence of *HOX* expression yet they readily acquire a *HOX* code when co-cultured with Hox-positive MSC (Liedtke et al., 2013).

Pronounced stability of the *Hox* code in the postnatal period may be important for proper healing and tissue regeneration. *Hox* gene expression is locally enhanced at the site of healing cutaneous wound (Uyeno et al., 2001) or bone fracture (Rux et al., 2017), supporting the proposed importance of *Hox* gene expression in these processes. Indeed, *Hox* gene upregulation in the site of injury strongly correlates with the regeneration outcome. Recent work by Qu et al. (2020) on murine digit tip regeneration demonstrated that only the successful regeneration is accompanied by temporary upregulation of *Hoxa13* and *Hoxd13* genes – ones that regulate digit development in embryogenesis. Furthermore, *Hox* gene expression is critical for fibroblast-dependent mechanisms of wound healing. It was demonstrated by Hansen et al. (2003) using transgenic diabetic mice that exhibit diminished wound healing. Exogenous delivery of *Hoxd3* gene to wound bed by plasmid injections accelerated wound closure in these mice which was mediated by robust increase of collagen production by fibroblasts.

Data from in *vivo* experiments suggest that a mismatch between expression patterns of *Hox* genes in the graft and its surroundings may lead to decreased graft survival (Dani et al., 2017). This was accurately demonstrated in a model of bone regeneration after heterologous transplantation (Leucht et al., 2008). In this study, fates of Hoxa11-positive MSC from the tibia and Hox-negative neural crest stem cells from the mandibula were investigated after transplantation either to a Hox-positive (tibia) or Hox-negative environment (mandibula). It was established that transplantation of Hox-negative stem cells to a Hoxa11-positive region led to the acquisition of Hoxa11-positive status by transplanted cells followed by successful healing. In contrast, transplantation of Hox11-positive cells to a Hox-negative microenvironment resulted in a drastic reduction of bone tissue regeneration.

Thus, the contribution of *Hox*-code in resident stromal cells to tissue repair becomes a basis for further assumptions on potential objects and mechanisms to be investigated in this field. We should notice that the role of *Hox* gene expression in regeneration/repair is expected to be the most prominent in tissues with strong regeneration capacity which will be discussed below.

## Among MSCs, There Are Subpopulations Expressing *Hox*, and They Differ in Properties From Those That Do Not Express *Hox*

There is evidence that Hox-positive and Hox-negative subpopulations of stem or stromal cells may reside within the same tissue/organ. It was shown that these subpopulations, although residing in the same compartment, show distinct patterns of properties, for instance, specific *in vitro* differentiation abilities. Particularly, it has been shown that cord blood stem cells contain a subpopulation of USSC that do not express *Hox* and a pool of bone marrow-derived MSC that express *Hox* genes (*HOXA9*, *HOXB7*, *HOXC10*). This finding was concordant with unrestricted potency of USSC that can differentiate into cells of all three germ layers while cord blood MSC are limited to tissues of mesodermal origin (Liedtke et al., 2010). *Vice versa* bone marrow (HOX-positive) MSC, but not USSC (HOX-negative), readily undergo adipogenic differentiation in routine laboratory tests (Kögler et al., 2009).

Thus, it seems that the role of *Hox* gene expression in adult stromal and stem cells is not limited to storing positional information. Since *Hox* genes are master regulators of many processes, *Hox* code affects cell phenotype and, therefore, functional characteristics of a cell which allows claiming that Hox-positive and Hox-negative cells represent two distinct subpopulations. This is consistent with results (Bradaschia-Correa et al., 2019) that Hox status accurately defines transcriptomic differences and differentiation potential between periosteal MSC obtained from Hox-positive and Hox-negative anatomic sites. Recent findings by Rux et al. (2016) highlight a subpopulation of periosteal Hoxa11-positive MSC that was characterized as a progenitor-enriched subpopulation. The role of *Hox11* genes in bone repair will be discussed below supporting this remarkable finding by animal tests in a model of bone fracture.

Another example of the *Hox* gene that serves as a discerning marker within one anatomic region is *HOXC10*. The placenta contains both decidua-derived MSC in which *HOXC10* is highly expressed and amnion-derived MSC that lack its expression (Hwang et al., 2009). These two cell types demonstrate the dramatic difference in their potency and secretome composition (Kögler et al., 2004, 2005) supporting that presence or absence of a specific Hox may be used as a marker to discern between subpopulations of functionally different cells. Indeed, both MSC – from decidua and amnion – show a typical CD73/90/105 immunophenotype and fully comply with other criteria of MSC.

We have mentioned above that fibroblasts isolated from various regions of the body are characterized by different *Hox* codes reflecting their origin. This characteristic of cultured cells retains *in vitro* for a substantial period of time. However, this data was obtained by evaluation of *Hox* expression in a fibroblast culture after several passages (or after a 2-week colony formation assay), which raises a concern on whether a true physiological pattern of *Hox* expression can be reproduced in such kind of experiment. Isolation of MSC is a stress factor similar to tissue damage that affects the status of isolated cells including expression of key transcription factors. It was demonstrated that the phenotype of cultured MSC does not fully reflect the heterogeneity (including functional) of the cell population that existed *in situ* prior to isolation (Sacchetti et al., 2016). Indeed, selective conditions of culture medium may result in a proliferation of one subpopulation and loss of minor pools of cells or ones that fail to adapt to culture medium composition. Thus, over several passages, the composition and ratio of cellular subpopulations may change dramatically and where one may gain advantage another may be marginally eliminated. In addition, we have also mentioned that Hox-negative cells are able to “adopt” *Hox* expression patterns from surrounding cells both – *in vivo* and *in vitro* via an unestablished mechanism (Leucht et al., 2008; Liedtke et al., 2013). Therefore, it is possible that the entire primary culture, previously heterogeneous in *Hox* expression, may acquire a Hox-positive status over time *in vitro*.

## Resident Hox-Positive MSC Determine the Structure and Organization of Stroma

### Bone

Recent studies have revealed an important role of Hox11-positive stromal cells subpopulation in limb bone regeneration. During embryogenesis, *Hox11* paralogues regulate the development of bones in the forearms and lower legs (zeugopod). In the postnatal period, a subset of Hoxa11-positive MSC resides in these parts of the skeleton. After a fracture, the expression of *Hoxa11* drastically increases at the site of injury. It has been established that *Hox11* genes are necessary for successful fracture healing both in early stages when *Hox11* function is essential for maturation of chondrocytes and in later healing periods when remodeling of the extracellular matrix has been shown to be *Hox11*-dependent (Rux et al., 2016, 2017). *Hox11* genes play an important role not only in fracture healing but also in normal bone turnover: *Hox11*-expressing MSC regulate osteocyte renewal, promoting maturation of osteoblasts and maintaining natural spatial organization of collagen fibrils in the bone (Song et al., 2020).

Loss-of-function experiments show that bone MSC lacking expression of *Hox11* genes fail to completely differentiate to the osteogenic and chondrogenic lineages *in vitro* (Rux et al., 2017; Song et al., 2020). Bone marrow MSC isolated from different parts of the skeleton and therefore having a different *Hox* code also differ by the efficiency of adipogenic and osteogenic differentiation (Ackema and Charité, 2008). Thus, in MSC their *Hox* code does not only carry information about positional identity but also determines specific “bias” of multipotency in cells from different anatomical regions.

In mouse tibia, *Hoxa11* paralogues are expressed exclusively in a subpopulation of periosteal MSC with a PDGFRα+/CD51+/LepR+ immunophenotype (Rux et al., 2016) and it was established that these Hoxa11-positive MSC are essential for normal fracture healing (Rux et al., 2017) supporting that this subpopulation is functionally distinct from Hoxa11-negative MSC of the tibia.

Thus, Hox-positive MSC are necessary for both normal bone renewal and fracture healing. Moreover, we may speculate that during fracture healing Hox-positive MSC may temporarily induce *Hox* expression in other cell subpopulations as these characteristics of Hox-positive-to-Hox-negative cells crosstalk were described *in vitro* in multiple types of stromal cells from other tissue.

### Spleen

In recent study, Ueno et al. (2019) characterized a Hoxa11-positive subpopulation of MSC in neonatal mouse spleen. Experiments with ectopic transplantation of an embryonic spleen showed that MSC of this subpopulation differentiate into all three types of splenic stromal cells. After stroma formation, it undergoes repopulation by hematopoietic cells of the host finalizing ectopic spleen to a fully operational organ.

In humans, structural elements of the spleen also show a vivid potential to form heterotopically. There are reported cases of splenosis – a condition when autologous heterotransplantation of splenic cells occurs after the rupture of the organ’s capsule. Eventually, it results in ectopic formation of spleen tissue – typically in the abdominal cavity (Fremont and Rice, 2007). It is likely that HOXA11-positive MSC of the spleen may be crucial for the development of splenosis in humans via creating an ectopic stromal harbor for other cell types to build organ *ex situ*. This claim is supported by data in mice that Hoxa11-positive MSC can give rise to all types of spleen stromal cells.

Thus, *Hoxa11* gene expression may be a marker of MSC that function as splenic stroma organizers, and their potency to rebuild spleen’s stromal portion is a strong intrinsic feature realized via a specific *Hox*-dependent gene expression profile.

### Endometrium

Besides spleen, the human body has another structure that can “take root,” rebuild and function after autologous heterotransplantation – endometrium, the inner layer of the uterus.

Endometrium undergoes deep desquamation during every menstrual cycle and regenerates with a remarkable rapidity (within several days). This extraordinary ability of endometrium results in up to 200–300 cycles of complete regeneration over a woman’s lifespan and is mediated by specific properties of its stromal cells (endometrial MSC).

The capability of human endometrium to grow outside the uterus also underlies a serious condition known as endometriosis, in which endometrial tissue is ectopically formed in the abdominal cavity, on the surface of ovaries or even umbilicus. Endometriosis leads to hormonal disorders, infertility, and bleedings since ectopic tissue may undergo desquamation during the menstrual cycle (Vercellini et al., 2013).

Data on the potential role of *Hox* genes in endometrial renewal and ectopy are accumulated during recent decades and deserve an overview within the scope of this communication.

The development of the female reproductive system from the Mullerian duct is controlled by *HOXA9-HOXA13* genes. In particular, morphogenesis of the uterus is strongly regulated by *HOXA10* and *HOXA11*. In mice, impaired function of any of these genes leads to defects in a part of the reproductive system which they control during development (Taylor, 2000). In human development dysregulation of *HOXA10* expression may occur under the influence of a nonsteroidal estrogen medication – diethylstilbestrol (DES) and xenoestrogens, such as methoxychlor and bisphenol A. Both latter substances were widely spread for common purposes like protection of livestock from insect parasites (methoxychlor) or plasticware manufacture (bisphenol A). Influence of these xenobiotics on the developing embryo leads to abnormalities of the reproductive system mediated by persistent impairment of *Hox* gene expression (Ma et al., 1998; Fei et al., 2005; Smith and Taylor, 2007).

In the postnatal period *HOX* expression, specifically *HOXA10* and *HOXA11*, is retained in endometrium and in its resident MSC. Furthermore, there is a remarkable feature of endometrial MSC in which expression of *HOXA10* and *HOXA11* is regulated by steroid sex hormones: estrogen and progesterone.

**FIGURE 1:**
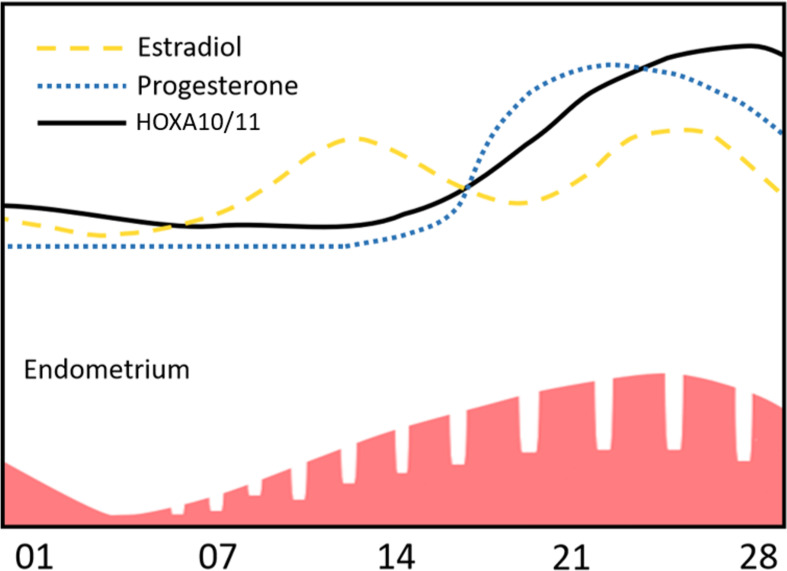
*HOXA10* and *HOXA11* expression dynamic is concordant to changes of estradiol and progesterone levels during the menstrual cycle.

During the menstrual cycle, plasma concentrations of estrogen and progesterone vary to regulate the switch of its phases. Expression of *HOXA10* and *HOXA11* in endometrial MSC also changes concordantly with undulations of hormone concentration (Figure 1). Expression of *HOXA10* and *HOXA11* factors is relatively low in the proliferative phase but increases and reaches its peak in the secretory phase and persists throughout menstruation (Taylor et al., 1998; Tang et al., 2006).

After menstruation, an extensively vascularized wound surface is formed. Menstrual discharge blood has decreased coagulation leading to the absence of granulations and rapid epithelisation followed by formation of the endometrial stroma. The latter is possible due to the proliferation and differentiation of endometrial MSC accompanied by intensive vascularization of tissue layers. We hypothesize that increased *HOXA10* and *HOXA11* expression in MSC during menstruation is an evolutionary established response required for quick and efficient regeneration.

Decidualization and embryo implantation that require the adequate function of the endometrium are *Hox*/*HOX-*dependent processes as well (Du and Taylor, 2015). Subsequently, most reviews of *HOX* genes’ role in endometrial function mainly focus on the relation of *HOX* family to female fertility.

We would like to draw attention to another aspect of *HOX* gene expression that can be stipulated as a putatively pivotal role of HOX-positive stromal cells in regeneration of the endometrium. It should be noted that the female reproductive system is one of the few regions in the adult organism that is characterized by a relatively high basal *HOX* gene expression compared to other body parts (Taylor, 2000). We suppose that elevated basal *HOXA* expression in the endometrium may reflect a specific higher threshold for enhanced control of regeneration and retainment of cell program during multiple cycles of its renewal throughout life.

No genetic disease caused by mutations of the *HOXA10*/*11* genes is known in humans, but there is evidence that a number of diseases of the female reproductive system such as hydrosalpinx, polycystic ovary syndrome and endometriosis are accompanied by decreased expression of *HOXA10* and *HOXA11* in the endometrium. Remarkably numerous and accurate studies show this for endometriosis (Wu et al., 2005; Du and Taylor, 2015). We want to pay special attention to the relation of impaired expression of *HOXA10* and *HOXA11* to endometriosis since this disease involves the ability of the endometrial stroma to self-organize ectopically, i.e., to “ignore” conditions of an ectopic environment.

We suggest that decreased expression of *HOXA10*/*HOXA11* in endometrial stromal cells may be a causal factor in the development of endometriosis. According to data on the interaction between a graft and a host with different *Hox* codes, lack of *Hox* expression in the transplant allows it to successfully engraft – either in a Hox-positive or Hox-negative host environment. Therefore, decreased expression of *HOXA10*/*HOXA11* genes results in partial loss of *HOX* code “identity” in endometrial cells facilitating its ectopy. It is of particular interest that the suppression of *HOXA10* results in increased autophagy proteins (beclin-1 and LC3-II) expression in endometrial tissue (Zheng et al., 2018). We suppose that it reflects a protective mechanism for clearance of cells with “loss of identity”, and that its failure results in the survival of ectopic endometrial cells with suppressed *HOXA10*/*11*. Being moved into the abdominal cavity they may adopt local HOX conditions and by unknown means avoid intrinsically activated autophagy to form endometrial tissue in an ectopic location.

Due to findings on Hoxa11-positive periosteum MSC, it is known that *Hox* gene functioning in stromal cells is of great importance for maintaining the normal structure of the stroma. In patients with endometriosis, the architecture of eutopic endometrium is impaired, including increased surface epithelium heterogeneity and reduced endometrial thickness (reviewed in Sharpe-Timms, 2001), which can also be caused or mediated by altered *HOX* expression in stromal cells of the endometrium.

Thus, available data suggests that the expression of *HOXA* genes in MSC of the endometrium ensures its normal functioning and regeneration during the menstrual cycle. We suggest that a HOXA-positive MSC subpopulation in the endometrium is critical for controlling its physiological regeneration after damage, as well as for maintaining the normal structure.

## Conclusion

**FIGURE 2:**
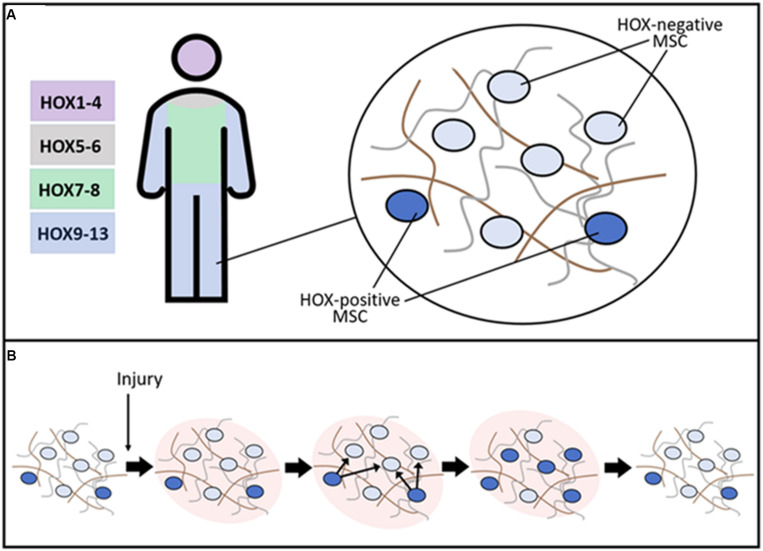
Putative role of resident HOX-positive MSC in the maintenance of stroma tissue-specific structure. **(A)** After embryogenesis, *HOX* gene expression is conserved in a distinct subpopulation of MSC. The schematic representation of *HOX* codes in distinct body parts is present. In normal tissue, a small HOX-positive subpopulation of MSC coordinates matrix turnover processes supporting normal architecture of stroma during physiological tissue renewal. **(B)** After damage, HOX-positive MSC are activated and induce expression of *HOX* genes in neighboring cells leading to the restoration of the initial structure of tissue stroma and facilitating regeneration.

Homeobox genes are critical during embryonic development of many animals. Expression of *Hox* is known to persist in many tissues in the postnatal period suggesting the role of these genes not only during development but also for the functioning of tissues throughout life. The tissue-specific pattern of *Hox* gene expression is inherent in stromal/stem cells of mesenchymal origin whose role in physiological renewal and regeneration is well-established in recent decades (Figure 2).

We believe that the generally adopted *Hox* code hypothesis and the role of these genes in supporting postnatal cell identity might lead us to a particularly important direction in the study of human regenerative biology. Key points of this hypothetical direction are summarized below:

1. In adult organisms, resident MSC represent a highly heterogeneous cell population with variable differentiation potency, sensitivity to regulatory stimuli, and genome expression. Recent studies demonstrated the existence of specific subpopulations of MSC necessary for the organization of normal tissue stroma with its tissue-specific features. Presumably, subpopulations of MSC retaining *Hox* expression may take specialized functions associated with the organization of the tissue-specific structure of the stroma. Our hypothesis relies on the expression of *Hox* genes as a specific feature of this subpopulation as far as resident MSC include cells of both types – highly expressing *Hox* genes and cells that lack *Hox* expression.
2. In humans, HOX-positive MSC subpopulations are crucial for maintaining and reconstructing stroma and have been identified within tissues that demonstrate a remarkable ability for regeneration (bone and spleen). We suggest that resident Hox-positive MSC are leading *organizers* of stroma renewal and tissue regeneration in other tissues as well and highlight a direction of research focused on the endometrium.
3. The peculiar feature of spleen and endometrium is ectopic growth which indicates a potent ability of cells within these tissues to rebuild a functional environment and give rise to organs (*e.g.*, splenosis). We suggest that *Hox* expression in a certain subpopulation of MSC in these tissues mediates their ectopic growth and efficient regeneration. Probably, identification of this subpopulation might be within reach in the endometrium as a feasible model object undergoing massive desquamation and reconstruction several hundred times. Data has been accumulated in favor of the assumption that Hox-positive stromal cells of the endometrium are important for its successful regeneration. Study of *Hox* patterns and their role in endometrial regeneration will allow more detailed insight into the functions of Hox-positive MSC and will expand our understanding of postnatal morphogenesis.

From a methodological point of view identification of crucial *Hox* genes using knockout or transgenic models may be a complicated mission due to functional redundancy of *Hox* paralogues. One may fail to exactly define an *in vivo* role of a given *Hox* using its knockout. To achieve a “loss of function” status with an obvious morphological outcome, a strain of animals with mixed knockouts and lacking several *Hox* genes may be required. In addition, conditional loss-of-function models are often obligatory – otherwise, it will be impossible to separate the effects of *Hox* disruption in embryonic development from defects that occur due to *Hox* gene suppression in the same structures in the postnatal period.

We expect our communication to trigger a certain amount of discussion and invite other authors and peers to comment on the potential of proposed direction of research and shall endeavor to decipher the role of *Hox* genes in regeneration using available models within our expertise (Eremichev et al., 2018; Nimiritsky et al., 2019).

## Author Contributions

MK: conception and drafting of the manuscript. PM: editing the manuscript, proofing, and funding acquisition. Both authors contributed to the article and approved the submitted version.

## Conflict of Interest

The authors declare that the research was conducted in the absence of any commercial or financial relationships that could be construed as a potential conflict of interest.
